# Proton Pump Inhibitors (PPIs)—An Evidence-Based Review of Indications, Efficacy, Harms, and Deprescribing

**DOI:** 10.3390/medicina61091569

**Published:** 2025-08-31

**Authors:** Monica Andrawes, Wessam Andrawes, Abhishek Das, Keith Siau

**Affiliations:** Royal Cornwall Hospital NHS Trust, Truro TR1 3LJ, UK; monica.andrawes@nhs.net (M.A.); wessam.andrawes@nhs.net (W.A.); abhishek.das3@nhs.net (A.D.)

**Keywords:** proton pump inhibitors, PPI stewardship, adverse effects, deprescribing, acid suppression, medication safety

## Abstract

Proton pump inhibitors (PPIs) are among the most prescribed drugs worldwide owing to their proven efficacy in symptom control and mucosal healing for acid-related disorders including gastroesophageal reflux disease (GORD), peptic ulcer disease, Helicobacter pylori eradication, functional dyspepsia, and gastroprotection in high-risk patients. However, long-term use beyond approved indications is increasingly common and has raised safety concerns. Observational studies link chronic PPI use to a myriad of adverse outcomes such as enteric infections (e.g., Clostridioides difficile), nutrient deficiencies (magnesium, vitamin B12), osteoporotic fractures, chronic kidney disease, dementia, and gastric and colorectal cancer. While causality is not always established, these associations warrant cautious risk–benefit assessment in patients receiving prolonged therapy. Current guidelines advocate periodic review of ongoing PPI use and emphasise deprescribing where appropriate. Strategies include dose reduction, on-demand or intermittent use, and switching to H2-receptor antagonists, particularly in patients with non-erosive reflux disease or functional dyspepsia. Tools from the National Institute for Health and Clinical Excellence, American College of Gastroenterology, and the Canadian Deprescribing Network assist clinicians in identifying candidates for tapering or discontinuation. This narrative review focuses on the concept of “PPI stewardship” by providing an evidence-based overview of PPI indications, risks, and deprescribing strategies to promote appropriate, safer, and patient-centred use of acid-suppressive therapy.

## 1. Introduction

Since their introduction in 1989, proton pump inhibitors (PPIs) have become increasingly used worldwide for both therapeutic and prophylactic indications [1,2,3]. PPIs inhibit the gastric H^+^/K^+^-ATPase enzyme system, the final step in acid secretion, offering more potent and sustained acid suppression than histamine-2 receptor antagonists (H2RAs) [4]. Their effectiveness and tolerability have established PPIs as some of the most frequently prescribed drug classes in primary care [5]. This phenomenon has been observed globally, reflecting increasing utilisation across multiple healthcare systems [6,7,8,9,10]. For example, >35 million PPI prescriptions were issued in the UK in 2022–2023, PPI use among adults in the USA reached ≈8.6% by 2017–2018, and approximately 2.87 billion PPI tablets were sold in Germany during 2020–2021 [7,11].

In Asia, national datasets highlight substantial PPI utilisation with high absolute volumes observed in China, particularly inpatient and injectable use between 2017 and 2021, while a sharp increase was seen in South Korea after ranitidine withdrawal (≈3929 doses/100,000/day) and growing but regionally heterogeneous PPI use in India [9,12,13].

There is growing concern with the widespread inappropriate initiation and prolonged duration of PPI therapy. A recent systematic review and meta-analysis estimated that up to 60% of PPI prescriptions lack a valid indication [14], a pattern observed globally among all age groups, including the geriatric population [14]. In a large UK primary care cohort, approximately one in four patients continued PPI therapy beyond 1 year; notably, many of these did so despite their initial indication not mandating long-term use and without evidence of dose-reduction attempts [15].

This overuse has drawn scrutiny from scientific and regulatory bodies due to increasing observational data associating long-term PPI use with potential harm. While causality remains uncertain, the consistency and biological plausibility of certain findings have prompted regulatory agencies such as the U.S. Food and Drug Administration (FDA) and the European Medicines Agency (EMA) to issue safety warnings and encourage periodic reassessment of therapy [1,16,17].

Recognising the gap between evidence-based indications and real-world prescribing, recent guidelines emphasise the need for PPI stewardship, including regular review and deprescribing where appropriate. Tools such as the Beers Criteria [18], STOPP/START criteria [19], and recommendations from the National Institute for Health and Care Excellence (NICE) [3] and Choosing Wisely campaigns [20] support clinicians in reviewing PPI use regularly and deprescribing when appropriate.

This narrative review focuses on PPI stewardship and provides current evidence on approved and off-label indications, comparative efficacy, and the spectrum of risks associated with prolonged PPI use. We also aim to outline practical, guideline-aligned strategies for prescribing, monitoring, and deprescribing to support safe and effective PPI use in clinical practice.

## 2. Methods

This narrative review was compiled following a literature search of three primary databases: Cochrane Library, PubMed, and Embase from 1999 to 2025. Search terms included combinations of the following keywords: “Proton Pump Inhibitors,” “PPIs,” “gastroesophageal reflux disease,” “peptic ulcer,” “PPIs co-prescription,” “deprescribing,” “long-term PPI use,” “H2R blockers,” “cytoprotective agents,” and “potassium competitive acid blockers (PCABs).”

Priority was allocated to national and international clinical guidelines, such as the National Institute for Health and Care Excellence (NICE), British Society of Gastroenterology (BSG), and the American College of Gastroenterology (ACG), systematic reviews, meta-analyses, and randomised controlled trials. Observational studies were included when trial data were lacking or to address rare adverse outcomes.

Inclusion criteria comprised studies or guidelines reporting on PPI indications, efficacy, co-prescribing, adverse effects, or deprescribing. Exclusion criteria comprised paediatric populations, non-English publications, abstracts without full text, and studies without clinically relevant data.

## 3. Indications of PPIs

The indications of PPI therapy depend on the underlying condition, treatment duration, and goals of therapy (Table 1) [3,21,22]. In clinical practice, many acid-related disorders are initially managed with a finite course of PPIs (“treatment phase”) followed by reassessment. For example, uncomplicated gastro-oesophageal reflux disease (GORD) or peptic ulcer disease is typically treated with an 4–8 week course to achieve mucosal healing and symptom resolution [23]. After this induction period, clinicians should reassess the need for continued therapy. If symptoms have resolved and no high-risk features are present, PPIs can often be tapered, discontinued, or switched to on-demand use.

In contrast, “maintenance therapy” refers to long-term PPI use aimed at preventing relapse or complications and should be prescribed at the lowest effective dose or on-demand dosing. Continuous daily PPI therapy is indicated in patients with high-risk conditions such as severe erosive esophagitis (Los Angeles grade C/D), Barrett’s oesophagus, active peptic ulcer bleeding, recurrent ulcer disease, or Zollinger–Ellison syndrome. Conditions in which PPIs should be avoided to reduce the risk of long-term harms are listed in Table 2, in line with recommendations from the American Gastroenterology Association (AGA) [22].

### 3.1. Co-Prescribing of PPIs

NICE recommends routine PPI prophylaxis in individuals over 45 years of age receiving long-term NSAIDs for chronic conditions such as osteoarthritis and rheumatoid arthritis [25]. Evidence from large-scale observational studies and meta-analyses shows that concomitant medications substantially increase the risk of gastrointestinal bleeding; for instance, the risk is up to 12-fold with corticosteroids, 11-fold with spironolactone [26], and 7-fold with selective serotonin reuptake inhibitors (SSRIs) [27]. Preventative strategies combining PPIs with misoprostol and COX-2 inhibitors are highly effective in lowering the risk of symptomatic ulcers and severe gastrointestinal complications, whereas H2 antagonists appear ineffective in preventing gastric ulceration [25]. ACG advises long-term PPI therapy as a prophylactic measure for patients at high risk of NSAID-induced GI haemorrhage, namely individuals over 60 years old, those with a history of peptic ulcers, or those taking corticosteroids and anticoagulants. The mortality rate from GI bleeds in NSAID users is higher (21%) compared to non-users (7%), indirectly attesting to the importance of PPI co-prescription as a gastroprotective measure [28].

#### 3.1.1. Antiplatelet Therapy

PPIs are frequently prescribed with aspirin or clopidogrel to reduce the risk of gastrointestinal bleeding [29,30,31,32]. This is particularly important in patients with a history of peptic ulcer disease or gastrointestinal bleeding. Recommendations from major gastroenterology and cardiology societies (Table 3) consistently endorse PPI co-prescription for patients receiving dual antiplatelet therapy (DAPT), reflecting the high gastrointestinal bleeding risk in this group. PPI use is advised for those on single antiplatelet therapy if additional risk factors are present, including previous peptic ulcer, advanced age, concurrent use of NSAIDs, anticoagulants, or steroids, and *H. pylori* infection [33].

#### 3.1.2. Helicobacter Pylori with Antiplatelet Therapy

The European Society of Gastrointestinal Endoscopy (ESGE) and European Society of Cardiology (ESC) jointly recommend testing and eradication of *H. pylori* in patients with a history of peptic ulcer disease before starting long-term antiplatelet therapy, especially if dual antiplatelet therapy is planned to reduce the risk of gastrointestinal bleeding [34]. The Maastricht V/Florence Consensus Report further supports this approach, specifically advising testing and treating *H. pylori* in patients starting long-term aspirin, particularly those with a history of peptic ulcer [35]. American [30] and British societies [3] recognise *H. pylori* as a risk factor for GI bleeding in patients on antiplatelet therapy but do not explicitly recommend routine testing and treatment before initiating therapy.

##### Practical Take-Aways

While there is consensus that PPIs should be co-prescribed with dual antiplatelet therapy in patients at high risk of gastrointestinal bleeding [29,30,31,32], there is variability in the breadth of recommendations. The ESC advocates routine PPI use in all patients on dual antiplatelet therapy [32], whereas American societies such as the ACCF/ACG/AHA advise a more selective approach, targeting those with additional gastrointestinal risk factors [29]. In practice, local prescribing is also shaped by drug licensing, reimbursement policies, and formulary restrictions [34]. In patients with a history of peptic ulcer disease, testing for and eradicating Helicobacter pylori before starting long-term antiplatelet therapy is increasingly recommended in European guidance [34,35] and may reduce bleeding risk.

#### 3.1.3. Anticoagulants

PPI co-prescription should also be considered in patients on warfarin or direct oral anticoagulants (DOACs) to reduce the risk of gastrointestinal bleeding, particularly in high-risk individuals [36,37]. A retrospective study by Ray et al. demonstrated significant reduction in hospital admission for upper GI bleeding in patients receiving PPI co-prescription with warfarin or DOAC [36]. A systematic review and meta-analysis conducted by Ahn et al. [37] associated PPI co-prescription in DOAC users with lower odds of total [OR 0.67, 95% CI (0.62–0.74)] and major GI bleed [OR 0.68, 95% CI (0.63–0.75)]. The 2021 European Heart Rhythm Association (EHRA) Practical Guide recommends considering PPIs for patients on DOACs with risk factors for GI bleeding, without the need for anticoagulant dose adjustment [38]. Similarly, the ACC/AHA and the International Society on Thrombosis and Haemostasis (ISTH) support PPI use in patients with prior GI bleeding or ulcer disease but do not advocate routine co-prescription for all [30,39]. Although some pharmacokinetic studies suggest that PPIs, particularly pantoprazole, may modestly reduce dabigatran absorption, these effects are generally not clinically significant [40]. Pantoprazole is often preferred due to its minimal CYP450 interaction profile.

#### 3.1.4. NSAIDs

NSAID is one of the most commonly prescribed classes of drugs and one of the leading causes of peptic ulcers. In the United Kingdom, long-term use of NSAIDs causes peptic ulcers in 12–15% of patients. Statistics show that over 12,000 people are hospitalized each year due to NSAID-related upper gastrointestinal bleeding. This costs the NHS around GBP 500 million annually. On a global scale, NSAIDs contribute to over 100,000 deaths each year due to peptic ulcer and related complications. [28,41,42]. ACG recommends PPI co-therapy for patients at moderate to high risk of NSAID-associated upper GI bleeding, one or more risk factors: age >65 years, high-dose NSAID therapy, a history of ulcer disease, and concomitant use of aspirin, corticosteroids, or anticoagulants [28]. An international consensus recommends that for patients with a previous history of upper GI ulcer bleed who are required to be on NSAIDs, a cyclooxygenase-2 (COX-2) selective inhibitor should be preferred along with PPI co-prescription [43,44]. Similarly the NICE guidance in the United Kingdom advocates offering a gastroprotective treatment (such as PPI) for people who are being treated with NSAIDs, with due consideration to use this in the lowest effective dose for the shortest period of time and to review the risk factors and adverse events regularly [45,46].

#### 3.1.5. Corticosteroids

PPIs are commonly co-prescribed with systemic corticosteroids to prevent peptic ulcers, although there is limited evidence to support this in practice, which can contribute to overuse. Guidelines from different health organizations differ in their recommendations. NICE guidelines suggest considering PPIs for high-risk patients on corticosteroids, while ACG recommends PPIs for patients on long-term corticosteroids with additional GI risk factors, and the European League Against Rheumatism (EULAR) recommends gastroprotection with PPIs for patients on long term corticosteroids, especially when combined with NSAIDs or other risk factors [4,28,47,48].

## 4. Efficacy of Different PPIs

PPIs differ in their pharmacokinetic properties, onset of action, and potency of acid suppression. However, their efficacy in clinical practice in treating the common acid-related conditions are broadly comparable. The recognised factors which influence therapeutic outcomes include bioavailability, CYP2C19 metabolism, and duration of gastric acid inhibition (Table 4).

While all PPIs effectively treat GORD, peptic ulcer disease and H Pylori infection, there is variation in 24 h intragastric and oesophageal acid suppression which can affect efficacy in severe GORD, erosive oesophagitis, or in cases requiring high-dose or nocturnal acid relief. Dexlansoprazole offers strongest acid suppression due to its dual delayed-release formulation. On the other hand, pantoprazole is preferred where there is a high risk of drug interactions, polypharmacy, or in patients taking clopidogrel.

Comparative trials and meta-analyses (Table 5) consistently show that PPIs achieve superior healing rates and eradication outcomes compared with alternative acid-suppressive strategies. Moreover, recent evidence highlights the role of potassium-competitive acid blockers (e.g., vonoprazan) as emerging comparators, with some studies reporting superior efficacy in *H. pylori* eradication regimens.

### PPIs Dosage

PPI therapy may be categorised into low, standard, and high doses (Table 6). For most acid-related disorders, standard doses of PPIs are sufficient, while high-dose regimens are often reserved for short-term use in severe inflammation or refractory disease [3]. PPIs are prodrugs requiring acid-induced activation within the canaliculi of gastric parietal cells. As such, timing of PPIs in relation to meals is critical and should be aligned with maximal proton pump activity. The optimal timing of PPI use is morning dosing, typically 30–60 min before breakfast. Twice-daily dosing for conditions such as severe GORD and Zollinger Ellison syndrome could be prescribed, and doses are usually scheduled before breakfast and dinner. Once-daily evening dosing is less effective unless using dual-release formulations (e.g., dexlansoprazole), which provide extended acid suppression. An exception to fasting administration occurs during *H. pylori* eradication regimens, where PPIs may be given with meals and other antibiotics to improve compliance and adherence Pylori.

## 5. Potential Harms of Long-Term PPI Use

**Figure 1:**
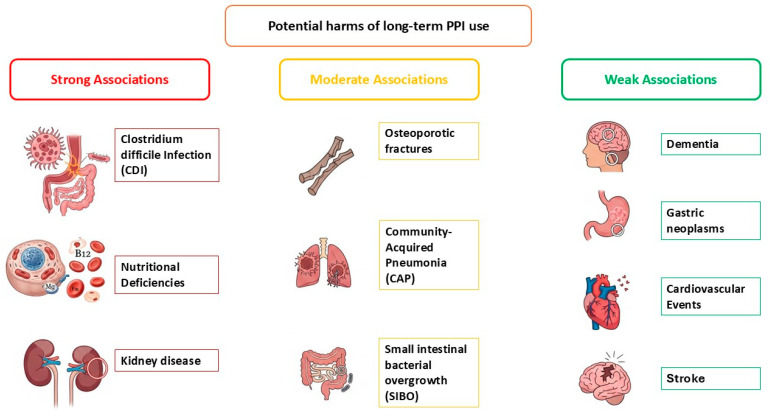
Potential harms of long-term use of proton pump inhibitors (PPIs).

The long-term safety of PPIs remains debated, with inconsistent findings across studies. For clinical decision-making, potential harms should be classified according to the level of evidence, strength of association linking PPIs to the adverse event, and biological plausibility. Adverse associations with long-term PPI use are summarised below and in Figure 1, whereas Table 7 summarises the main categories of PPI-associated harms, their estimated magnitude, and suggested monitoring strategies. Most available data are derived from observational studies rather than randomised controlled trials and should be interpreted with caution due to potential confounding.

### 5.1. Group 1: Harms with Strong and Consistent Association (Well-Established Risks)

#### 5.1.1. Clostridioide Difficile Infection (CDI) (Short-Term Risk)

PPIs are strongly associated with an increased risk of enteric infections, particularly *Clostridioides difficile* infection (CDI). The FDA has issued safety warnings citing studies demonstrating ~1.7-fold higher risk of CDI among users [59]. A recent systematic review and meta-analysis [54] indicated a positive association of CDI with PPI therapy, and an umbrella review (2025) confirmed this link across populations, finding pooled ORs ≈ 1.3–2.3 for CDI risk [60], although the dose–response relationship or threshold dosages remain unclear. The biological plausibility of this association is supported by two mechanisms: (i) PPI-induced hypochlorhydria reduces gastric clearance of ingested pathogens, increasing their survival and infectivity; and (ii) alterations in the gut microbiome (dysbiosis) create a milieu more conducive to colonisation and overgrowth of *C. difficile* and other enteric pathogens. PPIs are strongly linked to an increased risk of enteric infections. The FDA issued a warning based on studies showing a ~1.7-fold increased risk of *C. difficile*-associated diarrhoea [61]. A recent systematic review and meta-analysis [58] indicated a positive association of CDI with PPI therapy, and an umbrella review (2025) confirmed this link across populations, finding pooled ORs ≈ 1.3–2.3 for CDI risk [62], although the dose–response relationship or threshold dosages remain unclear. The plausible biological explanation includes increased intragastric pH, which reduces clearance of the organism and increases its infectivity and gastrointestinal dysbiosis, which leads to a milieu favourable to the growth of pathogenic organisms in the gut. These findings are from observational data, but the acid-suppression mechanism suggests PPIs likely contribute causally to CDI risk.

#### 5.1.2. Nutritional Deficiencies (Long-Term Risk)

Long-term use may lead to deficiencies in vitamin B12, magnesium, calcium, and iron due to impaired absorption in the setting of hypochlorhydria [63]. Reduced gastric acidity impairs the conversion of ferric to ferrous iron and the release of protein-bound vitamin B12, contributing to iron-deficiency anaemia and B12 deficiency in long-term PPI users [22]. A case-controlled Kaiser Permanente study found that long-term use (two years or more) of PPIs or H2RAs significantly increased the risk of vitamin B12 deficiency, particularly with high-dose PPIs. This reflects the need to consider potential B12 deficiency when prescribing acid-suppressing medications [57]. A meta-analysis of nine studies involving 109,798 patients revealed a higher risk of hypomagnesemia with PPI use (pooled risk ratio [RR] 1.43), with an even stronger correlation in cohort studies (RR 1.63) [55]. This has important implications for cardiovascular patients, in whom magnesium balance is critical and may lead to fatigue and neuromuscular irritation.

A retrospective cohort study of 98 adults on PPIs for at least one year indicated a decline in haemoglobin, haematocrit, and mean corpuscular volume compared to non-users. After adjustment, PPI users were over five times more likely to experience a significant drop in haemoglobin and haematocrit levels, raising concerns about the potential for iron deficiency anaemia with extended PPI use [58].These associations are observational, but they align with known physiology of acid-dependent nutrient absorption, supporting a likely causal link.

#### 5.1.3. Kidney Disease (Long-Term Risk)

PPI use is associated with an increased risk of acute and chronic kidney disease primarily due to acute interstitial nephritis [52,53]. A prospective cohort study involving 10,482 participants from Atherosclerosis Risk in Communities (ARIC) study and 248,751 patients from the Geisinger Health System showed that participants taking PPIs had a significantly higher risk of CKD incidence. PPI use was associated with a 24% and 50% increased risk of CKD in the Geisinger cohort and ARIC cohort, respectively, after adjusting for demographic, socioeconomic, and clinical factors. The risk was higher in participants on twice-daily PPI dosing [53]. A systematic review and meta-analysis of 10 observational studies (*n* = 6,829,905) revealed a 72% increased risk of developing CKD among PPI users compared to non-users [52]. These observational findings do not infer causation, although recurrent PPI-induced interstitial nephritis is a plausible mechanism. PPI use has been associated with an increased risk of chronic kidney disease and acute kidney injury [54,55]. The mechanism may involve recurrent acute interstitial nephritis. A prospective cohort study involving 10,482 participants from Atherosclerosis Risk in Communities (ARIC) study and 248,751 patients from the Geisinger Health System showed that participants taking PPIs had a significantly higher risk of CKD incidence. PPI use was associated with a 24% and 50% increased risk of CKD in the Geisinger cohort and ARIC cohort, respectively, after adjusting for demographic, socioeconomic, and clinical factors. The risk was higher in participants on twice-daily PPI dosing [54]. A systematic review and meta-analysis of 10 observational studies (n = 6,829,905) revealed a 72% increased risk of developing CKD among PPI users compared to non-users. However, further randomised controlled trials and biological studies are needed to confirm this association [55]. These are observational findings and do not establish causation, although recurrent PPI-induced interstitial nephritis is a plausible mechanism.

### 5.2. Group 2: Moderate Association

#### 5.2.1. Osteoporosis and Associated Fractures (Long-Term Risk)

Long-term PPI use has been linked with osteoporosis and increased fracture risk, with suggested mechanisms including calcium/B12 malabsorption, gastrin-induced parathyroid hyperplasia, and direct inhibition of osteoclastic vacuolar proton pumps [22]. While several prospective observational studies indicated reduced bone mineral density with long-term PPI use [61,62], prospective cohort studies by Targownik et al. [64,65] found no changes in bone mineral density or increased fracture risk. However, a systematic review and meta-analysis of 32 studies showed an increased risk of any-site fractures, hip fractures, spine fractures, and osteoporosis in PPI users [51]. These conflicting findings suggest observational associations that may be confounded; causality is not firmly established, and randomised trials show no clear detrimental effect on bone density. Therefore, caution should be exercised when prescribing PPIs, especially for the elderly, given the negative impact of bone fractures in this population. Long-term PPI use has been linked to osteoporosis and osteoporotic fractures, with suggested mechanisms including calcium/B12 malabsorption, gastrin-induced parathyroid hyperplasia, and osteoclastic vacuolar proton pump inhibition [25]. While several prospective observational studies indicated reduced bone mineral density with long-term PPI use [65,66], prospective cohort studies by Targownik et al. [67,68] found no changes in bone mineral density or increased fracture risk. However, a systematic review and meta-analysis of 32 studies showed an increased risk of any-site fractures, hip fractures, spine fractures, and osteoporosis in PPI users [56]. These conflicting findings suggest observational associations that may be confounded; causality is not firmly established, and randomized trials show no clear detrimental effect on bone density. Therefore, caution should be exercised when prescribing PPIs, especially for the elderly, given the negative impact of bone fractures in this population.

#### 5.2.2. Community-Acquired Pneumonia (CAP) (Short-Term Risk)

Short-term PPI therapy has been linked to an increased risk of community-acquired pneumonia (CAP) [56,66]. A systematic review of 33 studies analysed 226,769 CAP cases among 6,351,656 participants. The pooled OR for CAP with ambulatory PPI therapy was 1.49 (95% CI 1.16–1.92; I^2^ = 99.2%). Risk was highest during the first month of treatment (OR 2.10; 95% CI 1.39–3.16), independent of patient age or PPI dose. PPI therapy was also associated with greater risk of CAP-related hospitalisation (OR 1.61; 95% CI 1.12–2.31) [56]. The proposed mechanism involves hypochlorhydria-induced alterations of oral and gut microbiota, which may predispose to aspiration-related infection [66,67].

#### 5.2.3. Small Intestinal Bacterial Overgrowth (SIBO) (Long-Term Risk)

PPIs may increase the risk of SIBO, although the clinical significance is debated [68]. A systematic review and meta-analysis studied the connection between the use of PPIs and the likelihood of developing SIBO by pooling together 11 studies (3134 participants) and reported a positive association between PPI usage and an increased risk of SIBO OR 2.282). This association of a higher magnitude in studies that utilised duodenal or jejunal aspirate cultures (OR 7.58) was compared to studies centred on glucose hydrogen breath testing (OR 1.93) [69]. PPIs may increase the risk of SIBO, although clinical significance is debated [70]. A systematic review and meta-analysis studied the connection between the use of PPIs and the likelihood of developing SIBO by pooling together 11 studies (3134 participants) and reported a positive association between PPI usage and an increased risk of SIBO OR 2.282). This association was particularly notable in studies that utilized duodenal or jejunal aspirate cultures as a method (OR 7.587), but not in studies centred on the glucose hydrogen breath test (OR 1.93). These findings suggest that how SIBO is diagnosed could impact the observed connection between PPI use and SIBO [71]. These data are observational; the true clinical impact of PPIs on SIBO remains uncertain.

### 5.3. Group 3: Potential Risks (Weak/Uncertain Association)

#### 5.3.1. Dementia (Long-Term Risk)

Some cohort studies, including Haenisch et al. [72], found a modest association between PPI use and dementia (HR: 1.44, 95% CI: 1.36–1.52). However, subsequent studies and meta-analyses report inconsistent findings. Importantly, a 2023 prospective cohort study in adults ≥ 65 years of age found no association between PPI or H2RA use and incident dementia or cognitive decline. A 2024 Mendelian randomization study similarly found no robust causal link between PPI use and dementia risk [70,71]. These findings suggest that earlier associations were likely correlational and subject to confounding; the evidence does not support a causal relationship between PPIs and dementia [73].

Some cohort studies, including Haenisch et al. [73], found a modest association between PPI use and dementia (HR: 1.44, 95% CI: 1.36–1.52). However, subsequent studies and meta-analyses report inconsistent findings, Importantly, a 2023 prospective cohort study in adults ≥ 65 years of age found no association between PPI or H2RA use and incident dementia or cognitive decline. A 2024 Mendelian randomization study similarly found no robust causal link between PPI use and dementia risk [74,75]. These findings suggest that earlier associations were likely correlational and subject to confounding; the evidence does not support a causal relationship between PPIs and dementia [76].

#### 5.3.2. Gastric Neoplasms (Long-Term Risk)

A significant population-based study from Hong Kong [74] demonstrated that long-term PPI use after Helicobacter pylori eradication was associated with gastric cancer (HR: 2.44, 95% CI: 1.42–4.20), suggesting a dose–duration relationship. These results are from a retrospective cohort and do not establish causality; confounding factors like underlying gastric atrophy may contribute. More evidence is needed to determine whether PPIs independently raise gastric cancer risk.

#### 5.3.3. Cardiovascular Outcomes (Long-Term Risk)

PPIs may attenuate the antiplatelet effects of clopidogrel, especially omeprazole, potentially increasing cardiovascular events. Bhatt et al. [75] found no significant difference in serious cardiovascular events (HR: 1.02, 95% CI: 0.93–1.13); subgroup analyses and mechanistic data suggest caution, but current evidence does not demonstrate a harmful effect of PPIs on cardiovascular outcomes. Thus, any observed associations are likely non-causal; no definitive link with cardiovascular harm has been established.

PPIs may attenuate the antiplatelet effects of clopidogrel, especially omeprazole, potentially increasing cardiovascular events. Bhatt et al. [75] found no significant difference in serious cardiovascular events (HR: 1.02, 95% CI: 0.93–1.13). Subgroup analyses and mechanistic data suggest caution, but current evidence does not demonstrate a harmful effect of PPIs on cardiovascular outcomes. Thus, any observed associations are likely non-causal; no definitive link with cardiovascular harm has been established.

#### 5.3.4. Stroke (Long-Term Risk)

A Danish registry study found an increased risk of ischaemic stroke in PPI users, in particular with those taking higher doses and/or for a long duration [76], but causality remains uncertain. However, a 2025 systematic review and meta-analysis found only a modest association. Among patients without pre-existing cardiovascular disease, PPI use was associated with a slightly higher stroke risk (pooled HR ≈ 1.15, 95% CI ≈ 1.02–1.29) [77], whereas randomised trials in patients with CVD showed no significant increase (RR ≈ 1.158, 95% CI ≈ 0.914–1.466).

A Danish registry study found an increased risk of ischaemic stroke in PPI users, in particular with those taking higher doses and/or for a long duration [78], but causality remains uncertain. However, a 2025 systematic review and meta-analysis found only a modest association. Among patients without pre-existing cardiovascular disease, PPI use was associated with a slightly higher stroke risk (pooled HR ≈ 1.15, 95% CI ≈ 1.02–1.29) [79], whereas randomised trials in patients with CVD showed no significant increase (RR ≈ 1.158, 95% CI ≈ 0.914–1.466). These data indicate at most a weak observational link; there is no evidence of causality, and the overall stroke risk related to PPIs remains uncertain.

## 6. Overprescribing of PPIs

Studies suggest that 25–70% of PPI prescriptions lack an appropriate indication [5].

Overprescribing is particularly common in primary care and hospital settings. Contributing factors include prescribing for non-specific abdominal symptoms without proper diagnostic workup, inappropriate stress ulcer prophylaxis in non-critically ill patients, failure to de-prescribe after resolution of the initial indication, and perception of PPIs as completely safe medications.

Studies suggest that 25–70% of PPI prescriptions lack an appropriate indication [80]. Overprescribing is particularly common in both primary care and hospital settings. Contributing factors include use for non-specific abdominal symptoms without appropriate diagnostic work-up, inappropriate stress-\–ulcer prophylaxis in non-critically ill patients, failure to discontinue after resolution of the initial indication, and the perception of PPIs as entirely safe medications.

### 6.1. Evaluation for Discontinuation

Long-term PPI use is often excessive, exposing patients to avoidable costs and potential risks. Deprescribing (dose reduction, on-demand use, or discontinuation) should be routinely considered once the initial treatment course is completed. Patients who have completed appropriate short-term therapy for reflux or ulcer disease should be reviewed and stepped down if no compelling indication remains. UK guidance (e.g., NICE, NHS) recommends reassessing the need for therapy at least annually, with a trial of discontinuation where appropriate. The American Gastroenterological Association (AGA) also supports periodic review, with particular emphasis on tailoring continuation to the underlying indication.

#### Conditions to Avoid Discontinuation

Key contraindications to deprescribing include Barrett’s oesophagus, severe esophagitis (LA grade C/D), documented recurrent GI ulcer/bleeding, Zollinger–Ellison or eosinophilic oesophagitis with confirmed PPI response.

### 6.2. Different Guideline Recommendations

#### 6.2.1. NICE (UK CG184, 2014/2019)

NICE (UK CG184, 2014/2019): Emphasises using the lowest effective dose and on-demand therapy. If symptoms recur, it is recommended to step down to the lowest dose and discuss on-demand use. Annual review is advised, with trial of step-down or discontinuation if safe. Patients should be advised on alternative symptom control measures, including antacids, alginate preparations, or lifestyle modifications. H_2_RAs are recommended where PPI response is inadequate [3].

#### 6.2.2. AGA/ACG (2022)

AGA/ACG (2022): Recommends reviewing the indication for all PPI users. Patients without a clear ongoing indication should be offered a trial of deprescribing. Those on twice-daily dosing should usually be stepped down to once daily. It is not routinely deprescribed in complicated GORD (severe oesophagitis, strictures), Barrett’s oesophagus, eosinophilic oesophagitis, idiopathic pulmonary fibrosis, or patients at high gastrointestinal bleeding risk. Risk assessment tools are advised before withdrawal. Both gradual tapering and abrupt cessation are acceptable. The AGA stresses that decisions should be based on clinical indication, not concerns over potential side effects [22].

#### 6.2.3. Canadian (2017)

For adults with resolved reflux symptoms after ≥4 weeks of PPI, dose reduction or switching to on-demand therapy is strongly recommended. Alternatively, substitution with an H_2_RAs may be considered (weak to moderate evidence). Deprescribing is not advised in Barrett’s oesophagus, severe oesophagitis, or in patients with a history of bleeding ulcers [81].

**Figure 2:**
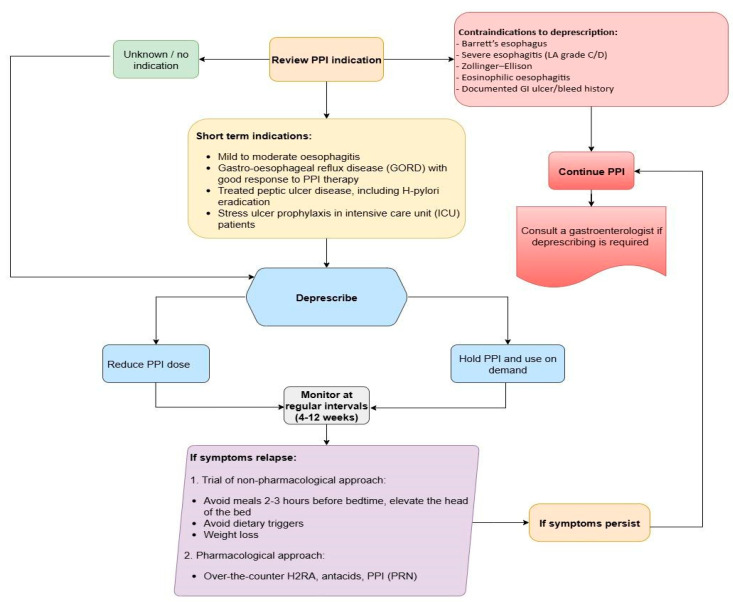
Algorithm for PPI evaluation and deprescription.

Based on these guidelines, we propose a flowchart for deprescribing evaluation (Figure 2).

### 6.3. Patient Groups and Deprescribing Actions

Uncomplicated GORD (mild reflux or NERD): Often suitable for tapering to the lowest dose or switching to on-demand therapy. Up to 80–85% of patients maintain remission with once-daily or on-demand dosing. Patients should be educated about rebound symptoms. Breakthrough can be managed with H2RAs or antacids [13,81].

Uncomplicated GORD (mild reflux or NERD): Often suitable for tapering to the lowest dose or switching to on-demand therapy. Up to 80–85% of patients maintain remission with once-daily or on-demand dosing. Patients should be educated about rebound symptoms. Breakthrough can be managed with H_2_ receptor antagonists or antacids [15,82].

Erosive oesophagitis: In mild to moderate disease (LA Grade A/B), step-down can be attempted. Severe oesophagitis (LA Grade C/D) warrants continued full-dose therapy due to high relapse risk. Endoscopic surveillance may guide treatment duration [13,81]. Erosive oesophagitis.

In mild to moderate disease (LA Grade A/B), step-down can be attempted. Severe oesophagitis (LA Grade C/D) warrants continued full-dose therapy due to high relapse risk. Endoscopic surveillance may guide treatment duration [15,82].

Barrett’s oesophagus: Generally, PPIs should not be stopped; long-term acid suppression is advised [22,81]. Barrett’s oesophagus: Generally, PPIs should not be stopped; long-term acid suppression is advised [25,26,27,28,29,30,31,32,33,34,35,36,37,38,39,40,41,42,43,44,45,46,47,48,49,50,51,52,53,54,55,56,57,58,59,60,61,62,63,64,65,66,67,68,69,70,71,72,73,74,75,76,77,78,79,80,81,82].

History of GI ulcer or bleeding: At least prophylactic therapy (for NSAIDs) should be maintained or full dose after a recent bleed. ACG explicitly lists prior bleed as a reason to continue PPI [25]. ACG specifically lists prior gastrointestinal bleeding as a reason to continue PPIs [22].

Helicobacter pylori–associated peptic ulcer: The standard 4–8 week PPI course should be completed alongside eradication therapy. Once healed, PPIs can be discontinued unless another indication exists. For patients requiring chronic NSAID or aspirin therapy, long-term PPI should be considered if gastrointestinal risk is high [78]. Helicobacter pylori–associated peptic ulcer.

The standard 4–8 week PPI course should be completed alongside eradication therapy. Once healed, PPIs can be discontinued unless another indication exists. For patients requiring chronic NSAID or aspirin therapy, long-term PPI should be considered if gastrointestinal risk is high [83].

NSAID/antiplatelet use: In patients on long-term NSAIDs or antithrombotics, PPI is indicated if gastrointestinal risk factors are present. Deprescribing can be considered if NSAIDs are stopped. Alternatives such as COX-2 inhibitors, non-pharmacological pain control, or other analgesics may reduce the need for PPI [34,78]. NSAID/antiplatelet use.

In patients on long-term NSAIDs or antithrombotics, PPI is indicated if gastrointestinal risk factors are present. Deprescribing can be considered if NSAIDs are stopped. Alternatives such as COX-2 inhibitors, non-pharmacological pain control, or other analgesics may reduce the need for PPI [34,83].

Stress ulcer prophylaxis (non-ICU): Should be reserved for ICU patients or those with major risk factors (e.g., mechanical ventilation, coagulopathy). Inappropriate use outside critical care remains common. PPIs should be discontinued on discharge or once the risk has resolved [5,22,81].Stress ulcer prophylaxis (non-ICU): 

Should be reserved for ICU patients or those with major risk factors (e.g., mechanical ventilation, coagulopathy). Inappropriate use outside critical care remains common. PPIs should be discontinued on discharge or once the risk has resolved [25,80,82].

Older adults may be more vulnerable to PPI-related adverse effects. Where no clear indication exists, a trial of deprescribing can be attempted with close follow-up and lifestyle modification (dietary changes, weight loss, head-of-bed elevation) [81]. 

### 6.4. Deprescribing Strategies

Deprescribing refers to the systematic reduction or cessation of PPIs in patients without a continuing high-risk indication. Given the potential harms of long-term PPI use, guidelines (e.g., NICE, ACG) recommend annual review of ongoing need and dose reduction or discontinuation where possible.

### 6.5. Example Scenarios

**Case 1**: A 70-year-old man aiming to reduce pill burden was started on omeprazole 4 years ago in primary care for heartburn but has been asymptomatic since initiation. He has never undergone endoscopy and appears to have only mild-to-moderate oesophagitis. There are no ongoing indications for long-term therapy (e.g., Barrett’s oesophagus, chronic NSAID use, or high-risk ulcer bleeding). He is therefore an excellent candidate for deprescribing. Given no evidence that one tapering approach is superior, the choice should be tailored to patient preference. A reasonable option is to discontinue and switch to on-demand therapy, with clinic follow-up at 4 and 12 weeks to monitor for recurrence of heartburn, dyspepsia, or regurgitation. If symptoms recur, management could include short on-demand PPI courses, OTC antacids, and lifestyle modifications.

**Case 2**: A 90-year-old woman in a long-term nursing home with dementia and prior stroke is unable to communicate symptoms reliably. She has been on long-term standard dose PPI therapy, but neither family members nor her records clarify the original indication. A prior hospitalisation 5 years ago for an unprovoked bleeding gastric ulcer is noted. PPI tapering to low dose could be considered, with cautious monitoring.

**Case 3**: A 62-year-old woman with persistent reflux despite deprescribing attempt. This patient has longstanding GORD treated with lansoprazole 30 mg daily. Attempts to stop resulted in recurrent reflux and a trial of famotidine for 4 weeks was ineffective. OGD revealed multiple fundic gland polyps. Management should include a shared discussion of options, including (a) continuation of lansoprazole at the lowest effective dose, (b) non-pharmacological strategies (dietary trigger identification and avoidance), and (c) low-dose on-demand PPI therapy.

## 7. PPIs Versus H2RAs, Gastroprotective Drugs and New Acid Suppressants

PPIs are typically preferred over cytoprotective agents and H2RAs according to European, British, and American guidelines. This preference is based on the superior effectiveness and safety profile of PPIs. Cytoprotective agents (e.g., misoprostol and sucralfate) and H2RAs are used in specific cases or when PPIs are inappropriate, either as alternatives or adjunctive therapy. New acid suppressants such as potassium-competitive acid blockers (PCABs) offer advantages over conventional PPIs. Vonoprazan, a PCAB, reversibly blocks the potassium-binding site of the proton pump, enabling faster and more consistent acid suppression [79]. It is acid-stable, food-independent, and accumulates in both acidic and neutral environments, maintaining sustained action without the need for activation [79]. It offers superior night-time acid control compared to PPIs [80] and maintains efficacy regardless of CYP2C19 polymorphisms, thereby reducing inter-patient variability [79,82] and is typically effective with once-daily dosing [83]. The following tables summarise key characteristics, pharmacological mechanisms, efficacy, safety, and clinical indications of acid-suppressive therapies, including PPIs, H2RAs, cytoprotective agents, and PCABs (Table 8). Additional tables compare their use across specific conditions (Table 9) and highlight the clinical relevance of PCABs versus traditional PPIs (Table 10). PPIs are typically preferred over cytoprotective agents and H2RAs according to European, British, and American guidelines. This preference is based on the superior effectiveness and safety profile of PPIs. Cytoprotective agents and H2RAs are usually used in specific cases or when PPIs are not appropriate, either as alternatives or adjunctive therapy. New acid suppressants such as potassium-competitive acid blockers (PCABs) offer advantages over traditional PPIs. Vonoprazan, a PCAB, reversibly blocks the potassium-binding site of the proton pump, enabling faster and more consistent acid suppression [84]. It is acid-stable, food-independent, and accumulates in both acidic and neutral environments, maintaining sustained action without the need for activation [84]. It offers superior night-time acid control compared to PPIs [85] and maintains efficacy regardless of CYP2C19 polymorphisms, thereby reducing inter-patient variability [84,86] and is typically effective with once-daily dosing [87]. The following tables summarise key characteristics, pharmacological mechanisms, efficacy, safety, and clinical indications of acid-suppressive therapies, including PPIs, H2RAs, cytoprotective agents, and PCABs (Table 8). Additional tables compare their use across specific conditions (Table 9) and highlight the clinical relevance of PCABs versus traditional PPIs (Table 10).

British guideline recommendations including indications, dosage, co-prescription, deprescribing, adverse events and alternatives are summarised in Table 11.

## 8. Clinical Questions, Recommendations and Stepwise Approach to Deprescribing

Who should receive a PPI? It should be restricted to patients with clear, evidence-based indications; reviewed annually [3,21,22,25,75]; restricted to patients with clear, evidence-based indications; reviewed annually.

When to co-prescribe? Should be co-prescribed with high-risk NSAID or antithrombotic therapy; local and international guideline thresholds should be followed [25,28,29,31,45].

What to monitor? Periodic checks of magnesium, vitamin B12, and iron in chronic users should be considered [55,57,58]; bone health in the elderly should be assessed [50].

When and how to deprescribe? Tapering to the lowest effective dose should be caried out or a switch performed to on-demand therapy once the acute indication resolves [22,81]; counsel should be held on possible rebound symptoms.

Special populations? Long-term therapy for Barrett’s oesophagus, severe erosive oesophagitis, and high GI bleed risk should be maintained [22].

When to co-prescribe? With high-risk NSAID or antithrombotic therapy; local and international guideline thresholds should be followed [27,29,32,35].

What to monitor? Periodic checks of magnesium, vitamin B12, and iron in chronic users should e considered [58,59,60]; bone health in the elderly should be assessed [52].

When and how to deprescribe? Tapering to the lowest effective dose should be carried out or a switch to on-demand therapy should be performed once the acute indication resolves [24,80]; counsel should be held on possible rebound symptoms.

Special populations? Long-term therapy for Barrett’s oesophagus, severe erosive oesophagitis, and high GI bleed risk should be maintained [24].

### Stepwise Approach to Deprescribing

Assess ongoing indication: Continue PPI at the lowest effective dose if a high-risk condition is present (e.g., severe oesophagitis LA C/D, Barrett’s oesophagus, Zollinger–Ellison syndrome, recent bleeding ulcer, or high-risk NSAID use).

Review resolution: If the acute condition has resolved, consider dose reduction or discontinuation.

Select tapering or step-down method: Options include dose reduction, on-demand therapy, or substitution with an H_2_ receptor antagonist or antacid.

Educate the patient: Warn about rebound acid hypersecretion (2–4 weeks) and offer management strategies.

Follow-up: Review symptoms after 4–12 weeks and adjust treatment accordingly.

Structured deprescribing programmes with patient education and follow-up can achieve up to 86% discontinuation success compared with historical rates of 30–60% [98].

In addition, deprescribing inappropriate long-term PPI use is likely to be cost-effective, with potential savings from reduced medication expenditure and avoidance of downstream adverse event costs, though formal economic evaluations remain limited [5].

## 9. Limitations

This review is a narrative synthesis and is thus subject to inherent limitations. We acknowledge bias in the coverage of UK and English-based guidelines. Prescribing practices and guidelines vary globally, so some recommendations may not apply equally in all settings (e.g., some countries have earlier access to PCABs or different guideline thresholds). We attempted to present a balanced view, but emphasis on certain topics (e.g., deprescribing strategies) reflects available literature and may underrepresent others. Finally, cost-effectiveness was considered outside the scope of this review and was not included.

## 10. Knowledge Gaps

Despite extensive observational data, causal links between PPIs and many reported long-term adverse outcomes remain uncertain. Further randomised controlled trials are needed to clarify the true risk of conditions such as chronic kidney disease [54,56], bone fractures [52], and infections [36,57]. The optimal deprescribing strategy—whether gradual taper or abrupt cessation—also requires stronger evidence [24,80]. Data are limited on the role of *H. pylori* testing before antithrombotic therapy outside Europe [30,35] and on the long-term safety and comparative efficacy of newer acid suppressants such as PCABs [84,86,91] in routine practice.

## 11. Conclusions

PPI stewardship is essential for minimising unnecessary exposure to potential harms while preserving symptom control. Routine evaluation of ongoing need must become standard practice, as studies reveal that up to 25% of patients continue with therapy for more than a year without reassessment and that 25–70% of long-term PPI prescriptions lack a valid indication [79], which may be ameliorated by the deprescribing and de-escalation strategies presented herein. Incorporating these guideline-aligned strategies and shared decision-making into routine care empowers patients, reduces the risk of potential side effects, and encourages responsible medication stewardship. To sustain these gains, future quality improvement initiatives should actively address PPI stewardship to ensure that all prescriptions have a clear indication and duration, similar to antimicrobial stewardship principles, ensuring that PPI therapy remains safe, appropriate and patient-centred.

## Figures and Tables

**Table 1:** Indications of PPI use.

Type of Indication	Long-Term PPI (>8 Weeks)		Short-Term PPI (<8 Weeks)	
	Definite	Conditional	Definite	Conditional
Therapeutic	GORD with erosive oesophagitis (LA C+D) Peptic stricture Eosinophilic oesophagitis with histological response Barrett’s oesophagus Zollinger-Ellison syndrome	GORD with incomplete response to short-term PPI GORD with recurrence of symptoms on PPI cessation Eosinophilic oesophagitis (maintenance) Idiopathic chronic cough (GORD-confirmed)	GORD *H. pylori* eradication (combination therapy) Non-erosive GORD (symptom relief) Peptic ulcer disease Mild peptic inflammation	Functional dyspepsia (trial) Laryngopharyngeal reflux (LPR) (trial and review) Mild gastritis
Prophylactic	- Chronic NSAID/aspirin use + high GI risk - Antiplatelet therapy post-bleeding ulcer - Systemic sclerosis with reflux	- Long-term corticosteroids + GI risk factors	- NSAID/aspirin use (short course + risk factors) - Post-endoscopic ulcer therapy - Stress ulcer prophylaxis (ICU only)	- Post-bariatric surgery (short-term) - Post-sclerotherapy and band ligation To cover short-term NSAID/high dose steroid prescription

PPI, Proton pump inhibitors; GORD, Gastro-oesophageal reflux disease; LA C+D, Los Angeles Classification, Grade C and Grade D; NSAID, Non-steroid anti-inflammatory drug.

**Table 2:** Conditions where PPIs should be avoided.

Conditions	Short-Term Use	Long-Term Use
Therapeutic	Isolated throat symptoms [24] Acute undifferentiated upper GI symptoms (e.g., pain, nausea, vomiting) not seem to be due to GORD or PUD [22] Isolated lower GI symptoms	Non-erosive reflux or functional dyspepsia with no response to high-dose PPI therapy Peptic ulcer disease including gastric or duodenal erosions
Prophylactic		Steroid therapy in the absence of concomitant NSAID/antiplatelet therapy [22] Prevention of recurrent GI bleed for reasons other than peptic ulcer disease [22]

**Table 3:** Summary of recommendation of different societies of PPI use with antiplatelet therapy.

Organization	Guideline Summary	Notes
ACCF/ACG/AHA (2010, updated 2016) [29]	PPIs are recommended for high-risk patients only on DAPT only. Routine use is not recommended.	Based on interpretation of COGENT trial results. Focussed approach; avoid blanket PPI use
ESC (2018) [32]	Routine PPI use for all patients on DAPT.	Emphasises reducing bleeding risks associated with DAPT.
International Consensus (2020) [30]	Conditional recommendation for PPI in patients with previous ulcer bleeding on DAPT.	Based on low-quality evidence, no increased mortality from DAPT and PPI.
ANMCO/AIGO (Italian Guidelines) [31]	PPI recommended for patients with GI risk factors, history of PUD, use of NSAIDs or steroids.	Focuses on additional risk factors including age, GORD, and dyspeptic symptoms.

**Table 4:** Different PPIs according to their properties.

PPI	% Time pH > 4 (24 h)	CYP2C19 Metabolism	Bioavailability (%)	Duration of Acid Suppression	References
Dexlansoprazole	~70–80%	Low (dual delayed-release bypasses metabolism impact)	~60–70%	~24 h (dual release)	[1,2]
Rabeprazole	~60–70%	Low (non-enzymatic metabolism largely)	~52%	~16 h	[25,28]
Esomeprazole	~60–70%	Moderate (less than omeprazole; S-isomer)	~64%	~16–20 h	[25,29]
Lansoprazole	~50–60%	High (significantly affected by CYP2C19 polymorphism)	~80–90%	~12–14 h	[25,29]
Pantoprazole	~45–55%	Moderate (less interaction than omeprazole)	~77%	~10–12 h	[28,29]
Omeprazole	~40–50%	High (extensively metabolized by CYP2C19)	~30–40%	~10–12 h	[25,28]

**Table 5:** Summary of key studies comparing PPI efficacy to alternative therapies.

Study (year)	Condition	Comparison	Key Finding	Reference
Wang et al., 2005 (meta-analysis)	Erosive oesophagitis	Standard-dose PPI vs. H_2_RA	PPIs healed significantly more patients at 2–8 weeks; PPI 2–8 wk healing > H2HRA at 8 wk (63.4% vs. 52.0%)	[49]
Jiang et al., 2024 (NMA)	*H. pylori* eradication	Vonoprazan-based vs. PPI-based	Vonoprazan triple (2 wk) had highest eradication rate; superior to PPI quadruple (RR ≈ 0.90 favoring vonoprazan)	[50]

**Table 6:** Proton pump inhibitor (PPI) dosage guide.

PPI	Standard Dose	Low Dose (On-Demand)	High (Double) Dose
Omeprazole	20 mg once daily (40 mg once daily if severe oesophagitis)	* 10 mg once daily (20 mg once daily if severe oesophagitis)	40 mg once daily (40 mg twice daily if severe oesophagitis)
Lansoprazole	30 mg once daily	15 mg once daily	* 30 mg twice daily
Pantoprazole	40 mg once daily	20 mg once daily	* 40 mg twice daily
Rabeprazole	20 mg once daily	10 mg once daily	* 20 mg twice daily
Esomeprazole Rabeprazole	† 20 mg once daily (40 mg once daily if severe oesophagitis)	Not available (2010 mg once daily if severe oesophagitis)	‡ 40 mg once daily (40 mg twice daily if severe oesophagitis)

Doses in brackets are specifically for use in severe oesophagitis; doses should be taken 30 min before breakfast and (if needed) 30 min before the evening meal; * off-label dose for GORD; † This is lower than the licensed starting dose in GORD, but is considered to be dose-equivalent to other PPIs; ‡ This dose is recommended for a double dose as the 20 mg dose of esomeprazole is considered to be equivalent to omeprazole 20 mg [3].

**Table 7:** Adverse effects of PPI therapy and suggested monitoring strategies.

Harm Category	Effect Size (95% CI)	Suggested Monitoring
Bone fracture (major)	RR ~1.28 (1.22–1.35) [51] RR ~1.28 (1.22–1.35) [52]	Ensure adequate calcium and vitamin D intake; consider bone density assessment in high-risk patients
Chronic kidney disease	HR ~1.26 (1.16–1.38) [52,53] HR ~1.26 (1.16–1.38) [54,55]	Monitor renal function (serum creatinine/eGFR) periodically
Community-acquired pneumonia	OR ~1.37 (1.22–1.53) [56] OR ~1.37 (1.22–1.53) [57]	Monitor for respiratory infections, especially early in treatment
Clostridioides difficile infection	OR ~1.26 (1.12–1.39) [54] OR ~1.26 (1.12–1.39) [58]	Review any new diarrhoeal illness promptly; avoid unnecessary long-term use
Hypomagnesaemia	OR ~1.78 (1.08–2.92) [55] OR ~1.78 (1.08–2.92) [59]	Check serum magnesium in long-term users, especially if on diuretics
Vitamin B12 deficiency	[57]	Check levels after >2 years’ continuous use or if symptomatic
Iron deficiency/anaemia	[58]	Assess iron status if unexplained anaemia develops during therapy

**Table 8:** Key differences characteristics between PPIs, H2RAs, gastroprotective drugs, and PCABs.

Characteristic	PPIs	H2RAs	Cytoprotective Agents	PCABs (e.g., Vonoprazan)
Mechanism	Irreversibly inhibit H+/K+ ATPase	Block histamine H2 receptors	Form barriers (sucralfate); prostaglandin analogue (misoprostol); antimicrobial (bismuth) [84,85,86]	Reversibly inhibit H+/K+ ATPase at potassium site [79]
Onset of Action	Slower onset	Faster onset [4]	Variable, often local [88]	Rapid [79]
Duration of Action	Long-lasting [4]	Shorter duration [4]	Requires frequent dosing [84,85]	Sustained, even during night-time [79,87]
Mucosal Protection	Indirect via acid reduction [89]	Indirect [89]	Direct mucosal protection [89]	Both acid suppression and mucosal healing
Adverse Effects	Long-term risks (e.g., *C. difficile*, fractures) [63] [64]	Headache, dizziness [2]	Diarrhoea (misoprostol), contraindicated in pregnancy [84]	Similar to PPIs, but with some rare serious AEs [88]
Drug Interactions	CYP2C19 interactions (esp. omeprazole) [90]	Fewer interactions [2]	Minimal	Lesser CYP2C19 influence [79,82]
Tolerance/Rebound	Rebound acidity after withdrawal [91]	Tolerance with prolonged use [92]	Not common	Minimal data; likely less tolerance development
Dosing	Once or twice daily	Once or twice daily	Multiple daily doses [84,85]	Often once daily [83]

**Table 9:** Different drug class usage according to specific indications.

Indication	Preferred Therapy	Key Points
GORD	PPIs first line	As per ACG/ESGE, H2RAs used for intermittent symptoms [21,93].
Peptic Ulcer Disease	PPIs preferred; H2RAs still effective	Famotidine prevents ulcers in aspirin users [94,95]
NSAID-Induced Ulcers	PPIs and misoprostol	Recommended in high-risk groups [28,96]
*H. pylori* Eradication	PPI-based regimens	Key in triple and bismuth quadruple therapy [35,93,97].

**Table 10:** Clinical relevance of PCABs versus PPIs.

Condition	Comparative Efficacy
GORD	Non-inferior/superior to PPIs; effective in erosive, non-erosive, and PPI-resistant disease [87].
*H. pylori*	Higher eradication in clarithromycin-resistant strains (65.8–69.6% vs. 31.9%) [50]
Ulcer Healing	Comparable to PPIs; faster symptom relief reported; rare serious adverse events possible [88]

**Table 11:** Summary of UK guidelines recommendations.

Aspect	UK Guidelines Summary	Notes
PPI Indications	PPIs are first-line treatment for GORD, peptic ulcer disease, and *H. pylori* eradication.	NICE and BSG recommend PPIs for GORD and ulcer healing.
Dosage and Duration	Standard dosing for GORD and ulcers. Long-term PPI use is common, especially in elderly patients.	Dosing varies based on severity; an annual review is recommended by the NHS to assess necessity.
Co-prescription with NSAIDs	NICE and BSG recommend co-prescription of PPIs with NSAIDs in high-risk patients (e.g., age > 65, history of ulcers, or anticoagulant use).	Reduces risk of GI bleeding, especially in patients on long-term NSAID therapy.
Co-prescription with Antiplatelet Therapy	PPIs should be prescribed for patients on dual antiplatelet therapy (DAPT) or single therapy with additional GI risk factors (e.g., history of ulcers, *H. pylori*).	Prevents gastrointestinal bleeding in high-risk groups.
Co-prescription with Anticoagulants	PPIs should be considered for patients on warfarin or direct oral anticoagulants (DOACs) at high risk of GI bleeding. Pantoprazole is preferred due to fewer drug interactions.	Particularly important for patients with prior GI bleeding or ulcer history.
Co-prescription with Corticosteroids	NICE recommends considering PPIs for high-risk patients on corticosteroids, particularly when combined with NSAIDs or other GI risk factors.	Focuses on reducing risk of peptic ulcers from corticosteroid use.
Deprescribing PPIs	NICE and NHS recommend annual reassessment of PPI use to reduce overprescription. PPIs should not be stopped in high-risk patients (GORD, Barrett’s oesophagusesophagus).	Gradual tapering or continued use depending on clinical need.
Adverse Effects	Long-term PPI use is associated with risks such as gastrointestinal infections (*C. difficile*), nutrient deficiencies (B12, magnesium), and bone fractures.	NICE and NHS advise caution with long-term use; alternatives considered when appropriate.
Alternative Therapies	Sucralfate and misoprostol are alternatives for ulcer treatment, especially when PPIs are not suitable.	Cytoprotective agents and H2RAs as alternatives.
New Acid Suppressants	Potassium-competitive acid blockers (PCABs) like vonoprazan may offer advantages over traditional PPIs, particularly in PPI-resistant GORD and *H. pylori* eradication.	Emerging therapies are considered, but PPIs remain standard.

## Data Availability

No new data were created or analysed in this study. Data sharing is not applicable to this article.
